# Congenital Pyloric Stenosis*Read before the Travis County Medical Society.

**Published:** 1916-06

**Authors:** Joe Gilbert

**Affiliations:** Austin, Texas


### Congenital Pyloric Stenosis.*

JOE GILBERT, M. D., AUSTIN, TEXAS.

This condition was fully described first by Hirschspring in 1888. Since that time there have been numbers of cases diagnosed, treated and described. There are three types of pyloric stenosis in infants: spasmodic, hypertrophic and the combined type. It is the hypertrophic type that I shall discuss.

The age of the patient is of great importance as a diagnostic for the reason a large per cent of cases occur not later than the age of six weeks. Some few have been described as occurring at the end of the first week. The greatest majority, however, occur from the third to the sixth week. Some cases have been known to occur after changing from mother's to cow's milk. In regard to sex, more males are affected than females.

The most generally accepted theory as to cause is that it is a congenital organic defect resulting from primary pathologic hypertrophy of pyloric wall which constricts the lumen, which is most plausible cause for the hypertrophic type of this disease. Other theories offered are that the essential lesion is not muscular but primarily nervous, or that the trouble is caused by erroneous feeding, or by hyperacidity. This theory would account for the purely spasmodic stenosis, and these are the cases that will respond to medicinal treatment. The typical pathologic picture would be dilated stomach, and in long standing cases dilated esophagus with thickening toward pylorus, markedly thickened and hypertrophied pylorus, this cartilaginous tumor being one to three centimeters in length and one and one-half to two centimeters in thickness. The lumen of the pylorus is practically closed and alimentary tract below pylorus is normal in appearance and empty.

The onset of condition is usually sudden and unexpected and

---

*Read before the Travis County Medical Society.

is characterized primarily by development of an unusually severe and persistent vomiting, coming on after the ingestion of food. In some instances, however, the child may take two or three feedings and then vomit all of it at once, the expulsion being forcible and explosive in character. Constipation always exists, the degree being guided by the amount of stenosis present. The appetite is good, but, because of lack of assimilation of the food, the loss of weight is very marked. Another sign present, in most cases, is the peristaltic wave which consists of a rounded, circumscribed elevation of the abdominal wall, a lump from one to three inches in diameter which forms at the left of the umbilicus, sometimes appearing to rise from the margin of the ribs and passes across the epigastrium to the right hypochondrium, where it disappears. In a few seconds the phenomenon is repeated.

The prognosis and treatment depend upon the age of the patient, duration of illness, degree of emaciation, and form of obstruction. A great many cases come to a surgeon so weak and emaciated as to make the prognosis very unfavorable from a surgical standpoint. Medicinal treatment should be carried out for a short time to ascertain whether condition is of a spasmodic or hypertrophic character.

Hutchinson states that he has seen pronounced congenital pyloric stenosis cured under the medicinal plan; also Pearse, Russell, Huebrer, Starck, Blach and Bendix. In a recent article in *The Annals of Surgery,* Walton states that the surgical treatment has been more effective. He says that he has had personal observation of 23 cases, 19 of which were treated by medical means, and of this 19 only 1 recovered. Whereas four treated surgically two recovered. Scudder says that the mortality under medical treatment is between 80 and 90 per cent. He says that as far as he has been able to determine no true tumor has yet been cured by medical treatment, and that no so-called medically treated case has ever been proven to have had the disease. He also quotes cases reported by Murphy, Morse and Wohlbach where tumor was found at autopsy after successful gastro-enterostomy. This case, together with one of Walton's own, leads him to the definite conclusion that, in spite of the perfect rest obtained by operation, there is no tendency for the tumor to improve. Walton's case died seven months after operation from enterocolitis, and he made a thorough post-mortem examination of stomach and pylorus.

He found the following condition: Pylorus markedly hardened and thickened, forming a tumor-like mass three-fourths inch in length. He found that the pylorus was impervious to passage of fluids. The pylorus on section showed an enormous hypertrophy of muscular coats, and the mucosa could be seen thrown into folds which were tightly packed together so as to occlude the lumen. From the duodenal aspect the pylorus showed a characteristic projection into duodenal cavity. Microscopic section of pylorus showed that the thickening of the wall was due to a great hypertrophy of the circular muscular coats. The condition, therefore, was characteristic both clinically and pathologically of congenital pyloric stenosis. The symptoms were progressive but were entirely relieved by surgical treatment. Although none of the food passed through the pylorus, and this latter structure was at complete rest, yet there was no alleviation of muscular hypertrophy. The thickening of the pylorus was as marked at post-mortem examination as at operation and was impervious to passage of fluids.

My own observation in the following case leads me to believe that surgical treatment is the only means of relieving these conditions of total obstruction by a tumor. I cannot see how any case as thoroughly obstructed as this one was could be relieved in any way but bv surgical intervention. I would think, from my observation in this case, that those who have recovered by medical means were of a milder degree or else spasmodic in character.

REPORT OF CASE.

Boy, six weeks old. Seen by me when five weeks old, giving history of having vomited his nursing for five or six days previous to my first examination. Mother had given castor oil and attributed his trouble to this. Treatment began with complete rest of stomach for twenty-four hours. Weak solutions of barley water were tried without success, being vomited. Gave saline enemas two or three times per day. Let him rest for twelve or fourteen hours and then began breast milk in small quantities at short intervals. He went nearly all day and at night vomited with expulsive force the entire day's nursing. At this time baby began to have characteristic peristaltic waves of stomach described by all authorities as typical. A diagnosis of pyloric obstruction was then made. Patient was sent to infirmary, given twenty-four hours' treatment by Murphy drip without success. Operation was then advised and consultation asked for. Dr. J. W. McLaughlin saw the case with me and agreed on immediate operation. I decided that a posterior gastro-enterostomy was the operation of choice, having just previously seen an interesting case operated upon at one of the large New York hospitals, the surgeon doing a pyloroplasty. Upon opening the abdomen by incision above and to right of umbilicus and examining stomach, which was ballooned up to the size of a child's head, filled with gas and almost filling entire abdominal cavity, extending below umbilicus and almost to crest of the ileum. The pylorus was white and shining in appearance, broken like cartilage about one inch in thickness and one and one-half inches in length, impervious to the escape of even the gases of the stomach. The duodenum was entirely empty and flat. After seeing the condition, a posterior gastro-enterostomy was at once decided upon. The jejunum was picked up and held with straight tenaculum forceps, the posterior wall of the stomach pulled through lesser omentum in the usual manner and a post-gas-cut was done without clamps, much difficulty being experienced on account of small size of smaller intestines, it being about the size of a small lead pencil and flat.

The abdominal wall was closed with three layers of stitches, plain catgut for peritoneum, No. 2 chromic catgut for fascia, and coarse linen continuous for skin. I called for silkworm gut for through and through sutures (which I usually use), but nurse had failed to sterilize any, so could not use, the operation being hurried.

The time of operation required about thirty minutes. Baby was returned to room and put upon continuous Murphy drip for several hours. Baby reacted nicely without much shock.

A few hours after operation baby was given sterile water by dropper, one-half to a full teaspoon at a time. In about twelve hours began to give mother's milk by a spoon. In about twenty-four hours began to nurse at short intervals. Got along nicely until about seventh day. I started to change dressing on noticing some discharge, and on removing adhesive and gauze dressing I saw the wound was beginning to pull apart. At this moment baby began to cry and strain severely and the wound opened and the intestines began to protrude. Rushed immediately to operating room, holding sterile towel tightly over opening until sutures and instruments could hurriedly be prepared. Under ether by a nurse I closed wound with through and through large silk sutures, going one-fourth inch from margin of incision. During this operation I noticed my gastro-enterostomy opening and found it in fine condition without any suppuration. The second operation was a great shock to the baby, but he slowly reacted and was put upon same treatment as in preceding. This taught me a lesson, in operating upon babies, that interrupted sutures should be used throughout—because of the uncontrollable straining the abdomen of a baby is subjected to, when by continuous straining and tenseness of the muscles under discomfort they involuntarily aggravate conditions. This will loosen any other form of suture.

The baby was sent home about third week in good condition, taking his nourishment well with only an occasional spitting, as is the case when any baby is overfed. Mother was very nervous and excitable and hard to keep her milk up to standard.

About a week after returning home he developed a common catarrhal condition prevalent in the city at the time, developed a cough and a gastro-enteritis, which gave him another backset. At this time wound entirely healed. Baby had slow convalescence but improved gradually, and is now two years old and in good health.

I am reporting this case not because the subject has not been thoroughly discussed and described, but because it is interesting to me and unusual. In the seventeen years of general practice, it is the first case of congenital stenosis that I have personally had to treat. Second, it is the youngest baby I have ever done an abdominal section on. Third, it teaches me to be careful in my diagnosis of continuous vomiting in young babies, and to be particular in making an early diagnosis.

